# Chronic kidney disease predictors in obese adolescents

**DOI:** 10.1007/s00467-021-05403-2

**Published:** 2022-02-24

**Authors:** Katarzyna Mackowiak-Lewandowicz, Danuta Ostalska-Nowicka, Katarzyna Zaorska, Elzbieta Kaczmarek, Jacek Zachwieja, Martin Witt, Michal Nowicki

**Affiliations:** 1 Department of Pediatric Nephrology and Hypertension, Poznan University of Medical Sciences, Poznan, Poland; 2 Department of Histology and Embryology, Poznan University of Medical Sciences, Poznan, Poland; 3 Department of Bioinformatics and Computational Biology, Poznan University of Medical Sciences, Poznan, Poland; 4 Department of Anatomy, Rostock Univ. Med. Ctr., Rostock, Germany

**Keywords:** Glomerular filtration rate, Filtration barrier, Obesity, Chronic kidney disease, Adolescents

## Abstract

**Background:**

Glomerular hyperfiltration, initiating development of obesity-related glomerulopathy, results in an enlargement of the glomeruli and unsealing of the filtration barrier. It can be followed by adaptive focal segmental glomerulosclerosis and chronic kidney disease (CKD). The aim of the study was to determine the expression pattern of lipid metabolism and selected kidney damage markers in obese adolescents and to identify potential factors which can predict CKD.

**Methods:**

The study group consisted of 142 adolescents with a BMI *z*-score > 2. Sixty-two healthy and normal-weight individuals served as controls. The factors associated with the rate of glomerular filtration in obese adolescents were assessed by linear regression methods using univariate and multivariate analyses. The risk of developing CKD was estimated using the Fisher’s exact test.

**Results:**

The study group was divided into “elevated,” “normal,” and “decreased” glomerular filtration rate (GFR) patients. Increased urine galectin-3 (Gal-3) concentration was diagnosed in all patients. “Decreased GFR” subjects expressed increased urine concentration of neutrophil gelatinase-associated lipocalin (NGAL) and daily megalin excretion. Thirty-nine study participants developed CKD. Increased uric acid (UA) concentration was associated with CKD development both in “normal” and “decreased GFR” patients. Additionally, in “normal” GFR patients, increased concentrations of cholesterol (Ch), triglycerides (TG), and NGAL were associated with CKD.

**Conclusions:**

Increased serum concentrations of Ch, TG, and UA and increased urine concentration of NGAL might predict CKD development in obese adolescents with normal and decreased GFR.

**Graphical abstract:**

A higher resolution version of the Graphical abstract is available as Supplementary information

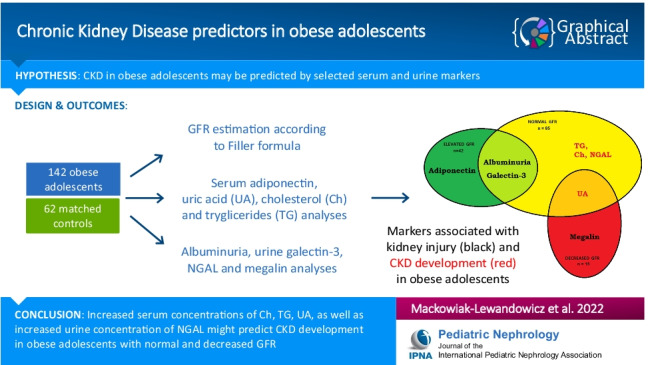

**Supplementary Information:**

The online version contains supplementary material available at 10.1007/s00467-021-05403-2.

## Introduction

Recent decades have revealed a significant change in the eating behavior and lifestyle of adolescents. These changes are mainly observed in the increasing consumption of high-calorie foods and sweet drinks, as well as in a more sedentary lifestyle and a significant lack of exercise [1]. The last approximately 2 years—during the COVID-19 pandemic—have only intensified this unfavorable behavior [2].

As a consequence, the steadily increasing global prevalence of overweight and obesity has reached epidemic proportions [3]. It is followed by systematically increasing incidences of obesity-related health effects in adolescents, such as cardiovascular diseases, type 2 diabetes mellitus, and obesity-related glomerulopathy (ORG)—defined as a distinct entity that includes proteinuria, glomerular enlargement, progressive glomerular sclerosis, and kidney function decline [4].

Obesity is a well-established low-grade inflammatory state reflected by the presence of circulating endocrine bioactive proinflammatory compounds and accompanied by reduced levels of anti-inflammatory adipokines [5]. The increased body mass, in its initial stage, also requires an increased blood perfusion through all organs, including the kidneys [10]. Such a hyperfiltration phase (as described in obesity-related kidney damage syndrome) leads to enlargement of the glomeruli (glomerulomegaly) and decreasing podocyte numerical density [6].

In animal models of ORG (Fisher rats ad libitum feeding), glomerulomegaly is followed by increasing cellular volume of podocytes, although at a lower rate than the increase in the volume of glomerular tuft [7]. As the podocytes form a post-mitotic population of cells, their ability to hypertrophy is limited, and the mechanical strain on these cells (resulting from stretch tension) reaches a breaking point. Individual podocytes fail and detach, causing localized denudation of the glomerular basement membrane and promoting microalbuminuria. It is then followed by subsequent adhesions to the Bowman capsule, forming areas for the development of segmental sclerosis [8].

The process of kidney damage in obese people is, however, more complex. The visceral adipose tissue may release into the circulation nonesterified fatty acids which can be accumulated in the liver, heart and kidneys [9]. In the case of kidneys, these compounds accumulate in mesangial cells, podocytes, and epithelial cells of proximal tubules [10, 11] and promote local inflammation and the expression of profibrotic growth factors [12].

The actual incidence of ORG is unknown. This is due to the fact that the undisputed diagnosis of ORG can only be made on the basis of a kidney biopsy (and these are performed less and less frequently) [4]. Moreover, ORG development—especially in its initial stages—is usually subclinical. The most important parameter indicating abnormal kidney function is microalbuminuria, which, however, can be easily overlooked [13]. This gives the illusion that obesity does not adversely affect kidneys. The situation is, however, the opposite. The progressive process of kidney sclerosis and fibrosis may lead to chronic kidney disease (CKD) and, finally, to kidney failure unnoticed [14–16]. The implementation of the treatment—weight reduction [17] and renin–angiotensin–aldosterone system (RAAS) blockade [14–16]—may be too late. For this reason, it seems important to detect obesity-related kidney reconstruction in a minimally invasive manner at the earliest possible stage and define markers that may indicate an increased risk of developing CKD in obese individuals.

Many attempts have been made to indicate the marker(s) of ORG progression. These include microalbuminuria [13], research on megalin, neutrophil gelatinase-associated lipocalin (NGAL), matrix metalloproteinases (MMPs), and tissue inhibitors of metalloproteinases (TIMPs)—regarded as acute kidney injury markers [18, 19]. However, we still do not have marker(s) that would not only allow the diagnosis of ORG, but also predict its course (e.g., obesity-related sclerotic and fibrotic processes in the kidney).

Galectin-3 (Gal-3)—a β-galactoside-binding lectin—has been shown to be involved in a number of biological processes including cell adhesion and activation [20], chemoattraction [21], cell growth and differentiation [20] as well as apoptosis [22]. Under normal conditions, it is primarily expressed in the proximal tubules and collecting ducts of the kidney [23]. Gal-3 is one of the most important proteins shown to be involved in renal fibrogenesis leading to CKD [24]. It is highly expressed and secreted by renal macrophages, which are usually detected in the glomeruli of all inflammatory glomerular diseases, but also in the interstitium of the kidney cortex and medulla [23]. The association of Gal-3 with the processes of cell adhesion and the regulation (progression) of inflammation suggests that this protein may also be up-regulated in the glomerular enlargement that accompanies the process of kidney hyperfiltration.

There are very few reports of Gal-3 in urine. Primarily its presence and clinical significance are described in blood serum. It is regarded as a sensitive diagnostic or prognostic biomarker for various pathological conditions including heart failure and cardiovascular diseases, oxidative stress, inflammation, and autoimmunity [20, 21, 25–27]. Therefore, the question that should be asked here is: Is it possible that Gal-3 (as a result of early kidney damage following hyperfiltration and glomerulomegaly) appears in the urine in diagnostic concentrations?

In line with the above, the aim of the current report was to:

1. Determine the expression pattern of lipid metabolism as well as kidney function markers (including Gal-3, megalin, MMP12, NGAL, and TIMP2) in obese adolescents with normal, increased, and decreased glomerular filtration rate (GFR).
2. Identify markers that may indicate an increased risk of developing CKD in obese adolescents.

## Patients and methods

### Ethical considerations

The protocol of the present study was approved by the Poznan University of Medical Sciences Local Bioethics Committee (resolution No 679/17). All investigations have been carried out in accordance with the Helsinki Declaration. The study participants and/or their legal guardians have given their written consent to participate in the study. Finally, the study has followed the standards of Good Clinical Practice.

### Design, place of study, and period

The study was conducted between 1 January 2018 and 30 June 2021 and involved 458 patients (recruited between 1 January 2018 and 31 December 2020), admitted to the Department of Pediatric Nephrology and Hypertension, Poznan University of Medical Sciences because of suspicion of arterial hypertension (single rise in blood pressure recorded by the school nurse). All subjects were hospitalized to perform 24 h arterial blood pressure monitoring (ABPM), echocardiography (ECHO), electrocardiography, and to perform laboratory tests: peripheral blood morphology (WBC, RBC, PLT, Hgb, HCT), serum creatinine, cystatin C, uric acid, urea, Na, K, Ca, Mg, total cholesterol, triglycerides, HDL, fasting glucose, TSH, fT3, fT4, liver enzymes (alanine transaminase, aspartate transaminase and gamma-glutamyl transpeptidase), total protein, C-reactive protein (CRP), adiponectin as well as 24 h urine for albumin, Gal-3, megalin, NGAL, MMPs and TIMPs.

As many as 224 subjects were not admitted to the study, as at least one of the following criteria was present: (1) age < 10 years or ≥ 16 years; (2) arterial hypertension; (3) genetic obesity or familial hyperlipidemia; (4) any aberrations in ECHO and electrocardiography; (5) hepatic dysfunction (serum concentrations of alanine transaminase > 40 U/l, aspartate transaminase > 40 U/l and gamma-glutamyl transpeptidase > 40 U/l); (6) autoimmune disease; (7) diabetes; (8) coexistence of neoplastic and/or any infectious disease; (9) morphological abnormalities in the urinary tract system; (10) renal hypotrophy (or a single kidney); (11) CKD; (12) kidney failure; (13) any complications of the perinatal period (including intrauterine growth restriction); (14) birth before the age of 36 weeks; (15) history of chronic disease; (16) history of oral contraceptive, antihypertensive, antidiabetic or lipid-lowering drugs as well as corticosteroids use; or (17) unsigned informed consent.

Arterial hypertension, according to the guidelines of the European Society of Hypertension [28], for teenagers < 16 years was defined as systolic blood pressure and/or diastolic blood pressure ≥ 95th percentile for sex, age, and height measured on at least 3 separate occasions.

### Study population

One hundred and forty-two adolescents were allocated for the study group. Sixty-two served as control subjects who met the following inclusion criteria: (1) age 10–16 years; (2) BMI *z*-score ≤ 2; (3) no arterial hypertension; (4) normal concentration of serum cortisol, glucose, thyroid hormones, and electrolytes; (5) no morphological abnormalities in the urinary tract system (including renal hypotrophy or agenesis); (6) GFR range between 90 and 130 ml/min/1.73m^2^; (7) absence of any chronic comorbidities; and (8) signed informed consent.

Patients enrolled in the study group had to meet the following inclusion criteria: (1) age 10–16 years; (2) BMI *z*-score > 2; (3) no arterial hypertension; and (4) signed informed consent.

With our available sample size, this study had 85% power for comparison of means in independent samples at the *p* < 0.05 level. The clinical and biochemical data in study subjects are summarized in Table 1.

**Table 1:** Clinical characteristics of adolescents from study and control groups

	Study group				Controls (*n* = 62)	*p*
	Elevated GFR (*n* = 42)	Normal GFR (*n* = 85)	Decreased GFR (*n* = 15)			
Demographic and anthropometric data						
Age [year]	14.1 ± 3.2	13.1 ± 3.4		13.5 ± 2.2	13.9 ± 3.8	ns
Male/female ratio	22/20	44/41		7/8	30/32	ns
BMI *z*-score	2.45 ± 0.81	2.68 ± 0.72		2.53 ± 0.77	0.72 ± 0.23	< 0.0001*
aBSI *z*-score	0.2 ± 1.1	0.2 ± 1.2		0.2 ± 1.0	0.05 ± 0.3	< 0.0001*
Selected analytical data: blood						
RBC (T/L)	4.4 ± 0.7	4.3 ± 0.7		4.2 ± 0.6	4.3 ± 0.5	ns
Hgb (g/dL)	13.4 ± 1.8	12.9 ± 2.4		13.1 ± 2.2	13.1 ± 1.5	ns
WBC (G/L)	6.9 ± 1.5	6.8 ± 2.1		6.4 ± 1.1	7.6 ± 1.1	ns
PLT (G/L)	273.3 ± 19.5	329.4 ± 28.2		299.1 ± 14.2	290 ± 19.3	ns
Creatinine (mg/dL)	0.77 ± 0.14	0.81 ± 0.19		0.82 ± 0.13	0.77 ± 0.11	ns
Uric acid (mg/dL)	3.9 ± 0.9	4.2 ± 1.1		6.1 ± 0.3	3.9 ± 1.4	0.004**
Cystatin C (mg/L)	1.1 ± 0.2	1.1 ± 0.3		1.0 ± 0.1	0.9 ± 0.2	ns
Glucose (mg/dL)	86.5 ± 5.4	84.2 ± 4.4		82.1 ± 2.2	87.9 ± 6.6	ns
Triglycerides (mg/dL)	143 ± 6.3	145 ± 9.8		169 ± 8.8	139 ± 8.3	0.037**
Cholesterol (mg/dL)	172 ± 7.2	164 ± 7.7		198 ± 5.4	166 ± 5.1	0.043**
HDL (mg/dL)	42.1 ± 4.5	45.3 ± 8.9		45.2 ± 2.2	48.2 ± 3.8	ns
Adiponectin (µg/ml)	4.7 ± 1.1	5.2 ± 2.3		4.6 ± 0.7	8.9 ± 1.9	0.005*
Selected analytical data: urine						
Albuminuria (mg/24 h)	17.2 ± 8.3	13.2 ± 7.2		19.2 ± 2.2	0	< 0.0001*
Gal-3 (ng/ml)	4.6 ± 2.4	4.7 ± 2.1		4.9 ± 1.7	2.3 ± 1.4	0.004*
Megalin (ng/24 h)	132.7 ± 33.2	141.2 ± 42.2		283.1 ± 12.2	141.4 ± 39.5	0.005**
NGAL (ng/ml)	4.3 ± 2.1	5.6 ± 3.2		12.7 ± 2.6	6.6 ± 1.5	0.005**
TIMP2 (ng/ml)	5.2 ± 3.7	4.9 ± 4.1		4.8 ± 2.6	4.3 ± 3.9	ns

*GFR*, glomerular filtration rate, *elevated GFR* GFR > 130 ml/min/1.73m^2^, *decreased GFR* GFR < 90 ml/min/1.73m^2^, *BMI* body mass index, *aBSI* a body shape index, *RBC* red blood cell count, *Hgb* hemoglobin, *WBC* white blood cell count, *PLT* platelets, *HDL* high density lipoproteins, *Gal-3* galectin 3, *NGAL* neutrophil gelatinase-associated lipocalin, *TIMP* tissue inhibitor of metalloproteinases

^*^As compared study to control adolescents

^**^As compared hypo-filtrated adolescents to controls

### Anthropometric measurements

Weight and height were measured using a certified columnar scale (SECA 799, Hamburg, Germany) with BMI function. BMI values were transformed into BMI *z*-scores using WHO reference values for pediatric BMI [29].

Waist circumferences were measured in centimeters using inextensible tape parallel to the ground. The measurement area corresponded to half the length between the lowest rib and the iliac crest, at the end of normal expiration. A body shape index (aBSI) was calculated by employing the following formula: aBSI = WC(m)/[BMI^2/3^ × H (m)^1/2^] [30]. It was then converted to age- and sex-specific *z*-scores calculated based on normative values of the US population [31].

### Blood and urine collection

A 5 ml whole blood was taken from all fasting adolescents in the morning between 7:00 and 8:00, after all-night rest on the first day of hospitalization. Half of the total blood was used for routine laboratory tests in all patients, and the rest of the blood sample was centrifuged. Serum with added BHT (10 ul 0.5 M BHT/1 ml sample) was frozen at – 80 °C and preserved for further analyses.

Laboratory tests in all patients also included first morning urinalysis and 24-h urine collection (for albumin, Gal-3, megalin, NGAL, MMPs and TIMPs). The 24-h urine amount was measured after thorough mixing. 10 ml of each urine sample was then centrifuged within 2 h after collection at 1,000 rpm for 10 min to remove cellular debris. The cleaned urine samples were stored at − 72 °C until next measurement.

### Glomerular filtration rate estimation

GFR was assessed by Filler formula involving serum cystatin C concentration, instead of plasma creatinine [32, 33]. Hyperfiltration was defined as GFR > 130 ml/min/1.73 m^2^. In contrast, a reduced GFR (hypofiltration) was defined as GFR < 90 ml/min/1.73 m^2^ [34].

CKD was defined as kidney damage or GFR lower than 60 ml/min/1.73 m^2^ for a period of at least 3 months [35].

### Biomarkers

Daily megalin excretion was estimated using Human LRP2/Megalin ELISA assay (LS-F11978-1, BD BioSciences, Warsaw, Poland). The concentrations of NGAL, Gal-3, MMP12, and TIMP2 were estimated using Human Lipocalin-2/NGAL Quantikine ELISA Kit (R&D Systems, Minneapolis, MN), Human LGALS3/Galectin-3 ELISA Kit (RAB0661, Sigma-Aldrich, Saint Louis, MO), Human MMP-12 ELISA Kit (P39900, Invitrogen, Carlsbad, CA), and TIM2 Human ELISA Kit (P16035, ThermoFisher Scientific, Waltham, US-MA), respectively.

Finally, serum adiponectin concentration was assessed with the use of Human Adiponectin ELISA kit (ab99968, Abcam, Cambridge, UK).

All samples from patients and controls were run in duplicate. The assays were performed according to the manufacturer’s instructions. The optical density (OD) value was determined twice, using a microplate reader, set to 450 nm and 540 nm as a wavelength correction. The readings at 540 nm were subtracted from the readings at 450 nm to correct for optical imperfections in the plate. The concentrations of NGAL, Gal-3, MMP12, TIMP2, and adiponectin were calculated based on the standard curve. These standards were also assayed in duplicate.

### Statistical analysis

All continuous variables are expressed as the mean ± SD. Statistical analysis was performed through the following steps: (1) Shapiro–Wilk test was used to determine the normal distribution of data; (2) one-way analysis of variance was employed to compare “elevated GFR,” “normal GFR,” and “decreased GFR” study subjects and controls; (3) Bonferroni correction was used as a general alpha adjustment for all of the multiple comparisons performed in the study; (4) non-parametric continuous variables were compared using the Kruskal–Wallis test; and (5) Pearson χ^2^ test was employed to analyze nominal data.

The independent factors for CKD development were determined using Fisher’s exact test. Generally, in Pearson correlation, a value of *r* greater than 0.7 was considered a strong correlation. Anything between 0.4 and 0.7 was a moderate correlation, and anything less than 0.4 was considered a weak or no correlation.

The factors potentially affecting the rate of glomerular filtration in obese adolescents (“serum uric acid concentration,” “serum triglycerides concentration,” “serum cholesterol concentration,” “serum adiponectin concentration,” “albuminuria,” “urine galectin-3 concentration,” “daily urine megalin excretion,” and “urine NGAL concentration”) were assessed using linear regression methods utilizing univariate and multivariate analyses. Regression coefficients (β) and 95% confidence interval (CI) for β were estimated for all linear models. The regression models were adjusted for age and sex.

All of the statistical analyses were performed using the Statistica 13.3 PL software package (StatSoft, Poland). A value of p < 0.05 was considered statistically significant.

## Results

### Clinical characteristics

Obese study participants (BMI *z*-score > 2), at the moment of entry into the analysis, were divided into “elevated GFR” (*n* = 42), “normal GFR” (*n* = 85), and “decreased GFR” (*n* = 15) according to GFR values estimated by Filler formula. As compared to controls, they presented albuminuria and had a significantly lower serum adiponectin concentration (*p* = 0.005) and higher urine Gal-3 concentration (*p* = 0.004). “Decreased GFR” obese adolescents (as compared to controls) have been additionally reported with significantly higher serum concentrations of uric acid (*p* = 0.004), triglycerides (*p* = 0.037), and cholesterol (*p* = 0.043) as well as significantly higher NGAL urine concentration and daily urine megalin excretion (*p* = 0.005).

Obese and control adolescents did not differ in blood pressure (both office and during 24-h monitoring), peripheral blood morphology, and serum biochemical parameters (including concentrations of creatinine, cystatin C, urea, Na, K, Ca, Mg, fasting glucose, TSH, fT3, fT4, liver enzymes, total protein, and CRP).

None of the examined adolescents had MMP12 in urine, and the concentrations of TIMP2 were comparable in all study participants. The clinical characteristics of all adolescents are summarized in Table 1.

### Correlations and multivariate analysis

As mentioned above, the group of obese adolescents was not homogeneous. It was composed of “elevated GFR,” “normal GFR,” and “decreased GFR” patients. Therefore, separate analyses were performed for each of the above-mentioned 3 subgroups.

In the “elevated GFR” obese study participants, we have noticed one moderate positive correlation between “urine galectin-3 concentration” and “albuminuria” (*r* = 0.528) and one moderate negative correlation between “urine galectin-3 concentration” and “serum adiponectin concentration.”

The group of “normal GFR” obese patients had a strong correlation between “urine galectin-3 concentration” and “urine NGAL concentration” (*r* = 0.706).

In the “decreased GFR” obese adolescents, three positive moderate correlations were reported. The first one between “serum uric acid concentration” and “urine NGAL concentration” (*r* = 0.474), the next one between “urine NGAL concentration” and “urine megalin excretion” (*r* = 0.524), and the last one between “serum uric acid concentration and urine megalin excretion” (*r* = 0.511). These results are presented in Table 2.

**Table 2:** Moderate and strong correlations in study participants

Pair of variables	*r*	*p*
“Elevated GFR” obese study participants		
Urine galectin-3 concentration and albuminuria	0.528	0.011
Urine galectin-3 concentration and serum adiponectin concentration	-0.422	0.033
“Normal GFR” obese study participants		
Urine galectin-3 concentration and urine NGAL concentration	0.706	0.001
“Decreased GFR” obese study participants		
Serum uric acid concentration and urine NGAL concentration	0.474	0.036
Urine NGAL concentration and urine megalin excretion	0.524	0.012
Serum uric acid concentration and urine megalin excretion	0.511	0.022

*GFR* glomerular filtration rate, *elevated GFR* GFR > 130 ml/min/1.73 m^2^, *decreased GFR* GFR < 90 ml/min/1.73 m^2^, *NGAL* neutrophil gelatinase-associated lipocalin

Estimates from linear regression models in study participants are shown in Table 3. Factors associated with obesity-related kidney damage in the period of hyperfiltration included decreased serum adiponectin concentration, albuminuria, and increased urine Gal-3 concentration. Factors associated with obesity-related kidney damage in obese adolescents with normal GFR were albuminuria, increased urine Gal-3 concentration, elevated serum cholesterol concentration, and increased urine NGAL concentration. Finally, obesity-related kidney damage followed by decreased GFR was associated with increased urine NGAL concentration, increased serum uric acid concentration, and increased urine megalin daily excretion (Table 4).

**Table 3:** The factors potentially affecting the rate of glomerular filtration in obese adolescents—linear regression analyses

	*β*	95% CI	*p*
	“Elevated GFR” obese adolescents		
Serum adiponectin concentration	-0.31	-1.08, 0.65	0.038
Serum triglycerides concentration	0.15	-0.02, 0.36	0.388
Serum cholesterol concentration	0.02	-0.01, 0.08	0.806
Serum uric acid concentration	0.11	-0.63, 1.45	0.460
Albuminuria	0.29	0.01, 0.79	0.042
Urine NGAL concentration	0.06	-0.02, 0.22	0.614
Urine galectin-3 concentration	0.35	0.06, 0.54	0.016
Urine megalin excretion	0.01	-0.01, 0.02	0.780
	“Normal GFR” obese adolescents		
Serum adiponectin concentration	-0.06	-0.23, 0.18	0.654
Serum triglycerides concentration	0.08	0.01, 0.36	0.580
Serum cholesterol concentration	0.30	-0.07, 0.87	0.022
Serum uric acid concentration	0.24	-0.05, 1.11	0.080
Albuminuria	0.32	-1.86, 1.89	0.038
Urine NGAL concentration	0.28	-0.01, 0.44	0.046
Urine galectin-3 concentration	0.31	0.01, 1.23	0.011
Urine megalin excretion	0.02	-0.02, 0.06	0.660
	“Decreased GFR” obese adolescents		
Serum adiponectin concentration	0.00	-0.01, 0.00	0.824
Serum triglycerides concentration	0.02	-0.01, 0.11	0.768
Serum cholesterol concentration	0.10	-0.02, 0.24	0.440
Serum uric acid concentration	0.28	0.02, 1.36	0.035
Albuminuria	0.15	-0.01, 0.38	0.380
Urine NGAL concentration	0.31	-0.01, 0.60	0.003
Urine galectin-3 concentration	0.16	-0.01, 0.33	0.111
Urine megalin excretion	0.34	0.01, 1.12	0.003

*GFR* glomerular filtration rate, *elevated GFR* GFR > 130 ml/min/1.73 m^2^, *decreased GFR* GFR < 90 ml/min/1.73 m^2^, *NGAL* neutrophil gelatinase-associated lipocalin

**Table 4:** Association of chronic kidney disease development in “normal GFR” and “decreased GFR” obese adolescents with initial serum uric acid, serum triglycerides, serum cholesterol, and urine NGAL concentrations based on Fisher’s exact test

	“Normal GFR” obese adolescents	“Decreased GFR” obese adolescents
Serum uric acid concentration	< 0.0001	0.0088
Serum triglycerides concentration	0.0006	-
Serum cholesterol concentration	< 0.0001	-
Urine NGAL concentration	0.0003	-

*GFR* glomerular filtration rate, *elevated GFR* GFR > 130 ml/min/1.73 m^2^, *decreased GFR* GFR < 90 ml/min/1.73 m^2^, *NGAL* neutrophil gelatinase-associated lipocalin

### CKD development in study participants

During the follow-up period, which ranged from 6 to 30 months, 39 obese adolescents in the study group developed CKD (27.5%). Eleven of them were initially designated as “increased GFR” individuals (26.2%), 16 as “normal GFR” study participants (18.9%), and 12 initially recognized with decreased GFR (80%).

"Elevated GFR" obese study participants, who developed CKD during the follow-up period, did not differ in peripheral blood morphology, serum creatinine, cystatin C, uric acid, urea, Na, K, Ca, Mg, total cholesterol, triglycerides, HDL, fasting glucose, TSH, fT3, fT4, liver enzymes, total protein, CRP, adiponectin as well as 24 h urine for albumin, Gal-3, megalin, NGAL, or TIMP2 values from their peers who did not develop CKD.

The majority of “normal GFR” obese study participants who developed CKD, were initially found with increased concentrations of serum uric acid (5.9 ± 0.6 mg/dL; *n* = 14; 87.5%), cholesterol (194 ± 5.8 mg/dL; *n* = 15; 93.8%), and triglycerides (172 ± 4.9 mg/dL; *n* = 10; 62,5%) as well as urine NGAL concentration (9.8 ± 3.5 ng/ml; n = 13; 81,2%), as compared to “normal GFR” obese adolescents who did not develop CKD.

Finally, “decreased GFR” obese adolescents who did not develop CKD within the observation period (*n* = 3) were found initially to have normal serum uric acid concentration (4.1 ± 0.3 mg/dL) as compared to their peers who developed CKD.

Using Fisher’s exact test, it was determined that “normal GFR” obese adolescents, who initially had elevated serum uric acid concentration (*p* < 0.0001), serum triglycerides concentration (*p* = 0.0006), serum cholesterol concentration (*p* < 0.0001), and urine NGAL concentration (*p* = 0.0003), developed CKD over a follow-up period of 6 to 30 months. “Decreased GFR” obese study participants who continue to have normal values of serum uric acid concentration are at lower risk of CKD development (*p* = 0.0088).

## Discussion

As mentioned above, the real incidence of ORG is unknown [4]. Since it is generally not possible to confirm ORG by histology, persistent albuminuria in obese individuals must be regarded as a sufficient indicator [16]. Other indicators of ORG, although not as common as subnephrotic proteinuria, are arterial hypertension, dyslipidemia, and decreased serum adiponectin concentration [36].

Our study group did not include adolescents diagnosed with hypertension. All of them were obese (BMI *z*-score > 2), and, according to GFR value estimated by Filler formula, they were divided into “elevated GFR,” “normal GFR,” and “decreased GFR” subjects. As expected, all of the study participants had albuminuria and decreased serum concentrations of adiponectin. However, the indicators of dyslipidemia (i.e., increased serum concentrations of cholesterol and triglycerides), as well as increased serum uric acid concentration, increased urine NGAL concentration, and increased megalin daily excretion, appeared only in “decreased GFR” adolescents. On the other hand, all of the study group participants (regardless of GFR value) had increased urine Gal-3 concentrations.

Interestingly, the presence of full symptomatic nephrotic syndrome in obese patients is distinctly unusual [14]. This may be due to the type of podocyte injury associated with the relatively rapid process of adaptive FSGS and glomerular fibrosis [37].

In our opinion, a marker that may explain the underlying cause of such kidney damage in obese individuals is Gal-3. It plays a crucial role in cell-to-cell adhesion [20], cell–matrix interactions [38], macrophage activation [21], as well as angiogenesis [39], metastasis [40], and apoptosis [22]. Interestingly, it has also been shown to be involved in the process of fibrosis in the liver [41], kidneys [42], and lungs [43] as the factor that stimulates fibroblasts, epithelial cells, and myofibroblasts to form collagen [44]. In particular, it has been recognized that Gal-3 is extremely useful for the detection of many of these diseases in their early stages. In experimental animal models using mice deficient in or lacking Gal-3, the process of fibrosis in any of the above-mentioned organs was limited or absent [45].

Obesity-related kidney damage, in the absence of therapeutic intervention, is evolving into CKD and, finally, to kidney failure unnoticed as a result of long-term hyperfiltration and accumulation of lipids in the kidney [14]. This is why we need a better (i.e., more specific) marker which will determine the risk of CKD in obese patients and sanction the correct (on time) implementation of RAAS blockade.

In the present report, 39 adolescents from the study group have developed CKD within the period of 6 to 30 months. Eleven of them were initially allocated to the “elevated GFR” group, 16 to the “normal GFR” group, and 12 to the “decreased GFR” group. Based on these allocations, we decided to determine the factors which might predict CKD in the subgroups noted above. In the “elevated GFR” study subjects, not a single marker was found to be associated with CKD development. In the “normal GFR” obese adolescents, however, 4 markers were found that could indicate CKD. These are increased serum concentrations of uric acid, triglycerides, and cholesterol, as well as elevated levels of urine NGAL. Finally, in the “decreased GFR” study participants, normal uric acid concentration was found as a factor protecting against CKD development.

The natural course of obesity-related kidney damage includes hyper-, normo-, and hypofiltration phases [14]. The second of these phases can be particularly troublesome. When determining GFR itself (and obtaining a value between 90 and 130 ml/min), we may not be aware of the fact that this is not a normal result, but a step leading directly to hypofiltration.

In the present study, an increased urine NGAL concentration in “normal GFR” obese adolescents strongly correlated with Gal-3. This observation may have clinically important implications. First, it indicates that NGAL is not only an indicator of acute kidney injury [46] but also of kidney remodeling. Second, it allows us to recognize those apparently “normal GFR” obese adolescents who are just before the hypofiltration phase. Third and finally, as mentioned above, it can be considered a useful predictor of CKD development in “normal GFR” subjects.

Several moderate (both positive and negative) correlations have also been shown between urine Gal-3 concentration and serum adiponectin and albuminuria (“elevated GFR” participants), as well as daily megalin excretion and NGAL urine concentration and serum uric acid concentration (“decreased GFR” subjects). These results suggest not only the key role of adiponectin in obesity-related kidney damage but also indicate megalin and NGAL as markers illustrating the progress of kidney injury in obese adolescents who have no arterial hypertension.

BMI *z*-score > 2 in children and adolescents corresponds to 97.7 percentile and indicates the risk of multiple organ damage, including kidney injury [29]. These subjects are not regarded as overweight individuals (who are between 85 and 97.7 percentiles; BMI *z*-score between 1 and 2); therefore, the weight loss and appropriate treatment should be initiated promptly. In numerous studies, introduction of angiotensin converting enzyme (ACE) inhibitors or angiotensin receptor blockers in obese patients with proteinuria or biopsy-proven ORG resulted in significant decrease of proteinuria [14–16]. What is more, ACE inhibitors demonstrated significantly greater antiproteinuric effect and the reduction in incidence of CKD and kidney failure in obese and overweight patients than in those with normal BMI [47]. For this reason, it seems to be extremely important to diagnose obesity-related kidney damage not only as quickly as possible, but also on the basis of more factors than subnephrotic proteinuria alone.

In line with the above, the implementation of dietary treatment and the introduction of ACE inhibitors in obese adolescents should be adapted to appropriate stage of disease defined not only by GFR and albuminuria, but also by the increased concentrations of urine Gal-3 and NGAL, daily megalin excretion, as well as increased serum uric acid concentration.

## Study limitations

Several limitations must be mentioned when discussing the results of the present study. First, it is a single-center study, so our cohort of 142 obese adolescents is not representative of the overall national population. Moreover, the participants were not randomly selected. Simply, all study subjects who met inclusion criteria were involved in the study protocol. Third, our study uses data that were collected two years ago and thus might not precisely reflect the current epidemiological situation.

None of the study participants had a kidney biopsy. For this reason, we were unable to recognize ORG despite the presence of subnephrotic albuminuria in any of them. This means that potential kidney remodeling in obese adolescents (which we described as obesity-related kidney damage) could only be identified indirectly.

We also present a relatively short observation period. Thus, we do not know how many patients would eventually develop CKD and kidney failure. The size of the “decreased GFR” obese adolescents group is relatively small, so it is difficult to draw an unambiguous conclusion that the normal concentration of uric acid may have a protective effect on the development of CKD.

## Supplementary Information

Below is the link to the electronic supplementary material.

Supplementary file1 (DOCX 31 KB)

Supplementary file2 (DOCX 31 KB)

Supplementary file3 (PPTX 76.3 KB)

## Data Availability

Data available upon request.

## References

[1] Kininmonth AR, Smith AD, Llewellyn CH, Dye L, Lawton CL, Fildes A (2021). The relationship between the home environment and child adiposity: a systematic review. Int J Behav Nutr Phys Act.

[2] Clemente-Suárez VJ, Dalamitros AA, Beltran-Velasco AI, Mielgo-Ayuso J, Tornero-Aguilera JF (2020). Social and psychophysiological consequences of the COVID-19 pandemic: an extensive literature review. Front Psychol.

[3] Tak YJ, Lee SY (2021). Long-term efficacy and safety of anti-obesity treatment: where do we stand?. Curr Obes Rep.

[4] Qorbani M, Khashayar P, Rastad H, Ejtahed HS, Shahrestanaki E, Seif E, Daniali SS, Goudarzi M, Motlagh ME, Khodaparast Z, Heshmat R, Kelishadi R (2020). Association of dietary behaviors, biochemical, and lifestyle factors with metabolic phenotypes of obesity in children and adolescents. Diabetol Metab Syndr.

[5] Hall JE, do Carmo JM, da Silva AA, Wang Z, Hall ME (2019). Obesity, kidney dysfunction and hypertension: mechanistic links. Nat Rev Nephrol.

[6] Chen HM, Liu ZH, Zeng CH, Li SJ, Wang QW, Li LS (2006). Podocyte lesions in patients with obesity-related glomerulopathy. Am J Kidney Dis.

[7] Fukuda A, Chowdhury MA, Venkatareddy MP, Wang SQ, Nishizono R, Suzuki T, Wickman LT, Wiggins JE, Muchayi T, Fingar D, Shedden KA, Inoki K, Wiggins RC (2012). Growth-dependent podocyte failure causes glomerulosclerosis. J Am Soc Nephrol.

[8] Kriz W, Hosser H, Hähnel B, Gretz N, Provoost AP (1998). From segmental glomerulosclerosis to total nephron degeneration and interstitial fibrosis: a histopathological study in rat models and human glomerulopathies. Nephrol Dial Transplant.

[9] Després JP, Lemieux I (2006). Abdominal obesity and metabolic syndrome. Nature.

[10] de Vries AP, Ruggenenti P, Ruan XZ, Praga M, Cruzado JM, Bajema IM, D'Agati VD, Lamb HJ, PongracBarlovic D, Hojs R, Abbate M, Rodriquez R, Mogensen CE, Porrini E, ERA-EDTA Working Group Diabesity (2014). Fatty kidney: emerging role of ectopic lipid in obesity-related renal disease. Lancet Diabetes Endocrinol.

[11] Bobulescu IA, Lotan Y, Zhang J, Rosenthal TR, Rogers JT, Adams-Huet B, Sakhaee K, Moe OW (2014). Triglycerides in the human kidney cortex: relationship with body size. PLoS ONE.

[12] Sun L, Halaihel N, Zhang W, Rogers T, Levi M (2002). Role of sterol regulatory element-binding protein 1 in regulation of renal lipid metabolism and glomerulosclerosis in diabetes mellitus. J Biol Chem.

[13] Serra A, Romero R, Lopez D, Navarro M, Esteve A, Perez N, Alastrue A, Ariza A (2008). Renal injury in the extremely obese patients with normal renal function. Kidney Int.

[14] Kambham N, Markowitz GS, Valeri AM, Lin J, D'Agati VD (2001). Obesity-related glomerulopathy: an emerging epidemic. Kidney Int.

[15] Praga M, Hernández E, Morales E, Campos AP, Valero MA, Martínez MA, León M (2001). Clinical features and long-term outcome of obesity-associated focal segmental glomerulosclerosis. Nephrol Dial Transplant.

[16] Tsuboi N, Koike K, Hirano K, Utsunomiya Y, Kawamura T, Hosoya T (2013). Clinical features and long-term renal outcomes of Japanese patients with obesity-related glomerulopathy. Clin Exp Nephrol.

[17] Friedman AN, Chambers M, Kamendulis LM, Temmerman J (2013). Short-term changes after a weight reduction intervention in advanced diabetic nephropathy. Clin J Am Soc Nephrol.

[18] Sandokji I, Greenberg JH (2020). Novel biomarkers of acute kidney injury in children: an update on recent findings. Curr Opin Pediatr.

[19] Nalesso F, Cattarin L, Gobbi L, Fragasso A, Garzotto F, Calò LA (2020). Evaluating Nephrocheck as a Predictive Tool for Acute Kidney Injury. Int J Nephrol Renovasc Dis.

[20] Dong R, Zhang M, Hu Q, Zheng S, Soh A, Zheng Y, Yuan H (2018). Galectin-3 as a novel biomarker for disease diagnosis and a target for therapy. Int J Mol Med.

[21] Suthahar N, Meijers WC, Silljé HHW, Ho JE, Liu FT, de Boer RA (2018). Galectin-3 activation and inhibition in heart failure and cardiovascular disease: an update. Theranostics.

[22] Funasaka T, Raz A, Nangia-Makker P (2014). Galectin-3 in angiogenesis and metastasis. Glycobiology.

[23] Ostalska-Nowicka D, Nowicki M, Kondraciuk B, Partyka M, Samulak D, Witt M (2009). Expression of galectin-3 in nephrotic syndrome glomerulopathies in children. Folia Histochem Cytobiol.

[24] Rebholz CS, Selvin E, Liang M, Ballantyne CM, Hoogeveen RC, Aguilar D, McEvoy JW, Grams ME, Coresh J (2018). Plasma galectin-3 levels are associated with the risk of incident chronic kidney disease. Kidney Int.

[25] Meijers WC, van der Velde AR, Ruifrok WP, Schroten NF, Dokter MM, Damman K, Assa S, Franssen CF, Gansevoort RT, van Gilst WH, Silljé HH, de Boer RA (2014). Renal handling of galectin-3 in the general population, chronic heart failure, and hemodialysis. J Am Heart Assoc.

[26] Fort-Gallifa I, Hernández-Aguilera A, García-Heredia A, Cabré N, Luciano-Mateo F, Simó JM, Martín-Paredero V, Camps J, Joven J (2017). Galectin-3 in peripheral artery disease. Relationships with Markers of Oxidative Stress and Inflammation. Int J Mol Sci.

[27] de Oliveira FL, Gatto M, Bassi N, Luisetto R, Ghirardello A, Punzi L, Doria A (2015). Galectin-3 in autoimmunity and autoimmune diseases. Exp Biol Med.

[28] Lurbe E, Agabiti-Rosei E, Cruickshank JK, Dominiczak A, Erdine S, Hirth A, Invitti C, Litwin M, Mancia G, Pall D, Rascher W, Redon J, Schaefer F, Seeman T, Sinha M, Stabouli S, Webb NJ, Wühl E, Zanchetti A (2016). European Society of Hypertension guidelines for the management of high blood pressure in children and adolescents. J Hypertens.

[29] Kêkê LM, Samouda H, Jacobs J, di Pompeo C, Lemdani M, Hubert H, Zitouni D, Guinhouya BC (2015). Body mass index and childhood obesity classification systems: a comparison of the French, International Obesity Task Force (IOTF) and World Health Organization (WHO) references. Rev Epidemiol Sante Publique.

[30] Krakauer NY, Krakauer JC (2012). A new body shape index predicts mortality hazard independently of body mass index. PLoS ONE.

[31] Krakauer NY, Krakauer JC (2016). An anthropometric risk index based on combining height, weight, waist, and hip measurements. J Obes.

[32] Filler G, Lepage N (2003). Should the Schwartz formula for estimation of GFR be replaced by cystatin C formula?. Pediatr Nephrol.

[33] Safaei-Asl A, Enshaei M, Heydarzadeh A, Maleknejad S (2016). Correlation between cystatin C-based formulas, Schwartz formula and urinary creatinine clearance for glomerular filtration rate estimation in children with kidney disease. J Renal Inj Prev.

[34] Schwartz GJ, Muñoz A, Schneider MF, Mak RH, Kaskel F, Warady BA, Furth SL (2009). New equations to estimate GFR in children with CKD. J Am Soc Nephrol.

[35] Levey AS, Eckardt KU, Tsukamoto Y, Levin A, Coresh J, Rossert J, De Zeeuw D, Hostetter TH, Lameire N, Eknoyan G (2005). Definition and classification of chronic kidney disease: a position statement from kidney disease: improving global outcomes (KDIGO). Kidney Int.

[36] Chen HM, Chen Y, Zhang YD, Zhang PP, Chen HP, Wang QW, Li LS, Liu ZH (2011). Evaluation of metabolic risk marker in obesity-related glomerulopathy. J Ren Nutr.

[37] Sethi S, Zand L, Nasr SH, Glassock RJ, Fervenza FC (2014). Focal and segmental glomerulosclerosis: clinical and kidney biopsy correlations. Clin Kidney J.

[38] Sciacchitano S, Lavra L, Morgante A, Ulivieri A, Magi F, De Francesco GP, Bellotti C, Salehi LB, Ricci A (2018). Galectin-3: one molecule for an alphabet of diseases, from A to Z. Int J Mol Sci.

[39] Blanda V, Bracale UM, Di Taranto MD, Fortunato G (2020). Galectin-3 in cardiovascular diseases. Int J Mol Sci.

[40] Kim SJ, Chun KH (2020). Non-classical role of Galectin-3 in cancer progression: translocation to nucleus by carbohydrate-recognition independent manner. BMB Rep.

[41] Moon HW, Park M, Hur M, Kim H, Choe WH, Yun YM (2018). Usefulness of enhanced liver fibrosis, glycosylation isomer of Mac-2 binding protein, galectin-3, and soluble suppression of tumorigenicity 2 for assessing liver fibrosis in chronic liver diseases. Ann Lab Med.

[42] Oikonomou T, Goulis I, Ntogramatzi F, Athanasiadou Z, Vagdatli E, Akriviadis E, Cholongitas E (2019). Galectin-3 is associated with glomerular filtration rate and outcome in patients with stable decompensated cirrhosis. Dig Liver Dis.

[43] Hirani N, MacKinnon AC, Nicol L, Ford P, Schambye H, Pedersen A, Nilsson UJ, Leffler H, Sethi T, Tantawi S, Gavelle L, Slack RJ, Mills R, Karmakar U, Humphries D, Zetterberg F, Keeling L, Paul L, Molyneaux PL, Li F, Funston W, Forrest IA, Simpson AJ, Gibbons MA, Maher TM (2020). Target-inhibition of galectin-3 by inhaled TD139 in patients with idiopathic pulmonary fibrosis. Eur Respir J.

[44] Yu L, Ruifrok WPT, Meissner M, Bos EM, van Goor H, Sanjabi B, van der Harst P, Pitt B, Goldstein IJ, Koerts JA, van Veldhuisen DJ, Bank RA, van Gilst WH, Silljé HHW, de Boer RA (2013). Genetic and pharmacological inhibition of galectin-3 prevents cardiac remodeling by interfering with myocardial fibrogenesis. Circ Heart Fail.

[45] Henderson NC, Mackinnon AC, Farnworth SL, Poirier F, Russo FP, Iredale JP, Haslett C, Simpson KJ, Sethi T (2006). Medical sciences galectin-3 regulates myofibroblast activation and hepatic fibrosis. Proc Natl Acad Sci.

[46] Kiryluk K, Bomback AS, Cheng YL, Xu K, Camara PG, Rabadan R, Sims PA, Barasch J (2018). Precision medicine for acute kidney injury (AKI): redefining AKI by agnostic kidney tissue interrogation and genetics. Semin Nephrol.

[47] Mallamaci F, Ruggenenti P, Perna A, Leonardis D, Tripepi R, Tripepi G, Remuzzi G, Zoccali C, REIN Study Group (2011). ACE inhibition is renoprotective among obese patients with proteinuria. J Am Soc Nephrol.

