# Supplementary material for: Chronic kidney disease predictors in obese adolescents
Source: Pediatr Nephrol. 2022 Feb 24;37(10):2479–88. doi: 10.1007/s00467-021-05403-2 (PMC8869344; doi:10.1007/s00467-021-05403-2)
Supplement: Supplementary file 3 — (PPTX 76.3 KB) [file 467_2021_5403_MOESM3_ESM.pptx]

## Slide 1
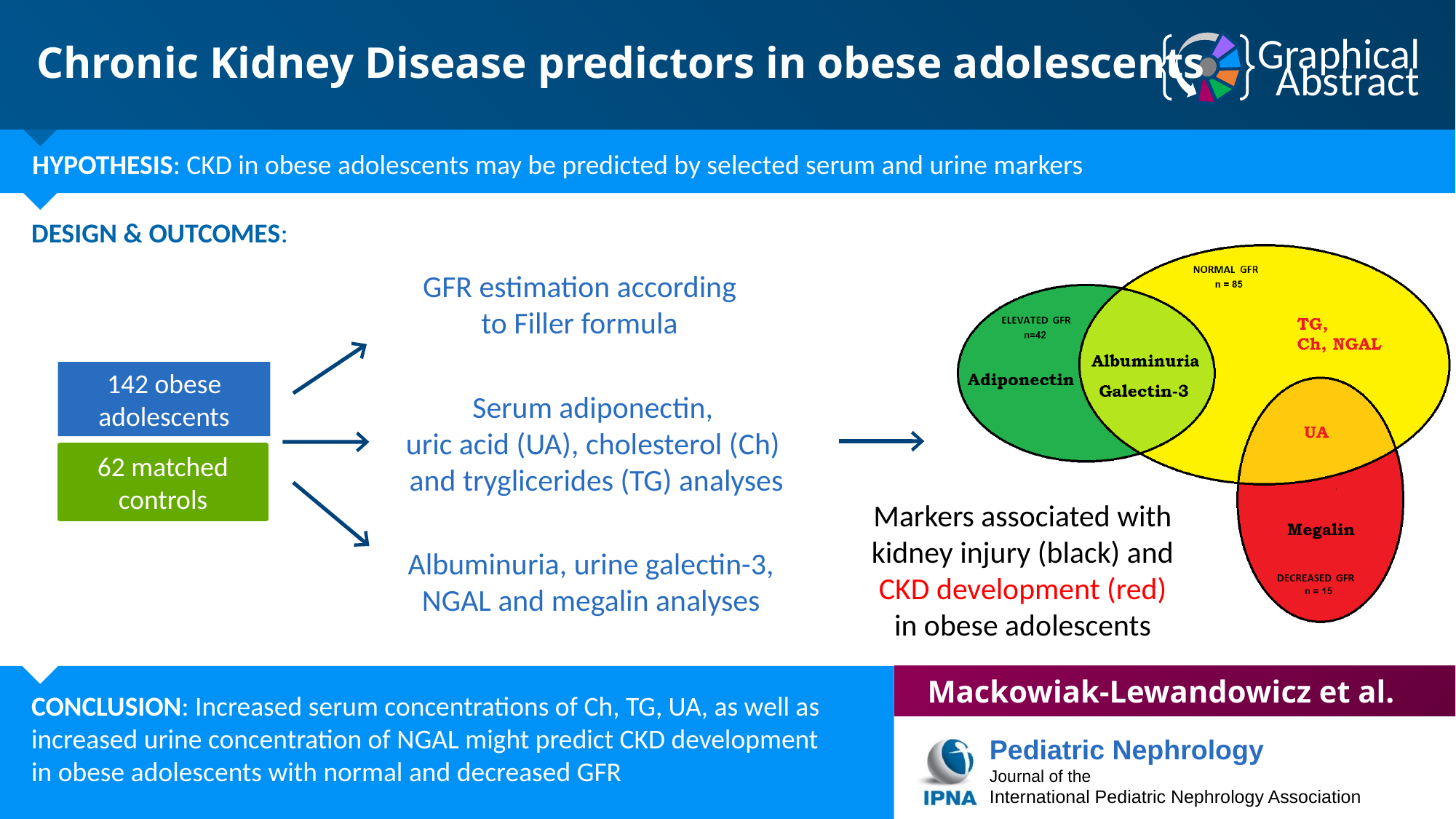

Chronic Kidney Disease predictors in obese adolescents
HYPOTHESIS: CKD in obese adolescents may be predicted by selected serum and urine markers
DESIGN & OUTCOMES:
GFR estimation according to Filler formula
142 obese adolescents
Serum adiponectin,
uric acid (UA), cholesterol (Ch)
and tryglicerides (TG) analyses
62 matched controls
Markers associated with kidney injury (black) and CKD development (red) in obese adolescents
Albuminuria, urine galectin-3, NGAL and megalin analyses
Mackowiak-Lewandowicz et al. 2022
CONCLUSION: Increased serum concentrations of Ch, TG, UA, as well as increased urine concentration of NGAL might predict CKD development in obese adolescents with normal and decreased GFR
